# Cardiac Health Risk Stratification System (CHRiSS): A Bayesian-Based Decision Support System for Left Ventricular Assist Device (LVAD) Therapy

**DOI:** 10.1371/journal.pone.0111264

**Published:** 2014-11-14

**Authors:** Natasha A. Loghmanpour, Marek J. Druzdzel, James F. Antaki

**Affiliations:** 1 Department of Biomedical Engineering, Carnegie Mellon University, Pittsburgh, Pennsylvania, United States of America; 2 Decision Systems Laboratory, School of Information Sciences, University of Pittsburgh, Pittsburgh, Pennsylvania, United States of America; 3 Faculty of Computer Science, Bialystok University of Technology, Bialystok, Poland; University of Louisville, United States of America

## Abstract

This study investigated the use of Bayesian Networks (BNs) for left ventricular assist device (LVAD) therapy; a treatment for end-stage heart failure that has been steadily growing in popularity over the past decade. Despite this growth, the number of LVAD implants performed annually remains a small fraction of the estimated population of patients who might benefit from this treatment. We believe that this demonstrates a need for an accurate stratification tool that can help identify LVAD candidates at the most appropriate point in the course of their disease. We derived BNs to predict mortality at five endpoints utilizing the Interagency Registry for Mechanically Assisted Circulatory Support (INTERMACS) database: containing over 12,000 total enrolled patients from 153 hospital sites, collected since 2006 to the present day, and consisting of approximately 230 pre-implant clinical variables. Synthetic minority oversampling technique (SMOTE) was employed to address the uneven proportion of patients with negative outcomes and to improve the performance of the models. The resulting accuracy and area under the ROC curve (%) for predicted mortality were 30 day: 94.9 and 92.5; 90 day: 84.2 and 73.9; 6 month: 78.2 and 70.6; 1 year: 73.1 and 70.6; and 2 years: 71.4 and 70.8. To foster the translation of these models to clinical practice, they have been incorporated into a web-based application, the Cardiac Health Risk Stratification System (CHRiSS). As clinical experience with LVAD therapy continues to grow, and additional data is collected, we aim to continually update these BN models to improve their accuracy and maintain their relevance. Ongoing work also aims to extend the BN models to predict the risk of adverse events post-LVAD implant as additional factors for consideration in decision making.

## Introduction

Cardiac transplantation currently represents the most definitive treatment for end-stage heart failure (ESHF) with 90% 1-year survival and a 70% 5-year survival. However, there is a need for alternate therapies due to the limited supply of donor organs. For those ineligible for a heart transplant, or unable to wait, an alternative life-sparing therapy is to implant a left ventricular assist devices (LVAD). These devices have been used for nearly 25 years to support ESHF patients while awaiting transplant and have been consistently shown to improve mortality. The technology has now progressed to the point where they are offered as permanent or so-called Destination Therapy (DT). According to current estimates, the number of ESHF patients who may benefit from LVAD therapy is between 80,000 and 200,000 annually. [1]

### LVAD Risk Scores

Optimal and responsible use of LVAD therapy requires a procedure for selecting patients who are most likely to benefit, and less likely to suffer adverse complications. In general, as a patient's disease progresses, the probability of poor outcomes increases. It is therefore important to identify candidates early in the progression of their disease so as not to miss the optimal window of opportunity [2], [3]. The window is considered between INTERMACS level 7 and 3, where 7 is clinically stable but history of previous decompensation and 3 is stable but Inotrope dependent [4]. This has motivated the development of risk scores to stratify patients based on the factors that have historically been associated with outcomes, such as patient characteristics, advances in mechanical circulatory support technology and surgical experience.

The most commonly cited score is the Lietz-Miller Destination Therapy Risk Score (DTRS), which was derived from a patient cohort with first generation pumps [5]. The first generation LVADs were pulsatile flow pumps, which attempted to mimic the physiological conditions. One-year survival in subjects undergoing the first generation pulsatile flow HeartMate XVE implantation for DT in the Randomized Evaluation of Mechanical Assistance for the Treatment of Congestive Heart failure (REMATCH) trial was 52% [6]. Enrollment criteria of initial studies emphasized hemodynamic variables. The DTRS analyzed 45 baseline parameters and outcomes in 280 DT patients in the post-REMATCH era. The most important determinants of in-hospital mortality were poor nutrition, hematological abnormalities, markers of end-organ and RV dysfunction and lack of inotropic support. Patients were stratified into low, medium, high and very high risk based on a score calculated from these predictors to correspond with 1-year survival [5]. The DTRS, however, has many limitations. The majority of patients in the derivation cohort were ambulatory, older men with large body surface area. Co-morbidities such as diabetes, cardiac cachexia or obesity were under-represented, while psychosocial factors or echocardiographic parameters were not considered.

Since the DTRS was derived, there has been major advances in the technology. In particular, second generation (continuous flow) pumps have become available that are smaller in size, have simpler technique for implantation, longer durability and present reduced risk of thromboembolism, infection and malfunction. Consequently, the frequency of adverse events has diminished, which has further expanded the candidacy pool for LVAD therapy. This has rendered the DTRS less accurate [7]. Other risk scores have been introduced, but are limited for a variety of reasons, such as limited independent variables or limited training data (e.g. from a single center.) For example, a recently introduced HeartMate II Risk Score (HMRS) relies on only five preoperative variables for predicting 90 day survival; the long-term (1 year) model only contained two: age and implant center experience [8].

### Clinical Decision Support Systems

The transition from paper to electronic medical records provides both a challenge and great opportunity for clinical decision making. On the one hand, the ever increasing quantity of information collected on a typical LVAD patient can overwhelm a clinical team, and arguably may introduce more uncertainty due to data incompleteness and noisiness. On the other hand, the wealth of information embedded in these data are ideal for computer-based Clinical Decision Support Systems (CDSS) [9], [10]. This is the motivation for developing the Cardiac Health Risk Stratification System (CHRiSS). CHRiSS is a web-enabled decision support tool that provides patient-specific predictions of mortality at 5 endpoints post-implant: 30 day, 90 day, 6 month, 1 year and 2 year. It offers several advantages over traditional LVAD risk scores. Since it is based on a Bayesian machine learning algorithm, it can better represent the influence of large sets of interrelated variables as compared to traditional Cox model multivariate predictors [11], [12]. Unlike most risk scores which compute survival at one time point, CHRiSS provides predictions of both short-term and long-term mortality. Since CHRiSS is implemented as an interactive software application, it also permits the user to explore various “what if” scenarios.

The purpose of this study is to evaluate the accuracy and sensitivity of the Bayesian Networks (BNs) in the CHRiSS tool. Accuracy is evaluated both in terms of True Negative (ability to predict survival) and True Positive (ability to predict mortality), which is also depicted by the receiver operating characteristics (ROC) curve. Sensitivity analysis was performed to identify the strongest predictive variables that are associated with either increased or decreased chance of survival post-LVAD.

## Methods

This study was conducted with a comprehensive dataset, known as the Interagency Registry for Mechanically Assisted Circulatory Support (INTERMACS). This is the largest national registry for U.S. Food and Drug Administration (FDA) approved mechanical circulatory support devices that is jointly sponsored by the National Heart, Lung, and Blood Institute (NHLBI), Centers for Medicare and Medicaid Services (CMS), FDA and industry. The registry has over 12,000 total enrolled patients (over 8,000 continuous flow LVADs) from 153 hospital sitesand has been collecting data since 2006 to the present day. The dataset consists of over 300 pre-implant clinical variables, subdivided into six main categories: demographics, co-morbidities and limitations from transplant listing, laboratory values, hemodynamics, medications and quality of life questionnaires and surveys. A co-morbidity in this context is defined as a medical condition or disease that exists simultaneously with another condition or disease. They can either be independent or related conditions or diseases.

### Patient Cohort

Institutional Review Board approval was obtained through hospitals participating within INTERMACS. The study described in this submission was approved by the INTERMACS Data, Access, Analysis, and Publication Committee (DAAP). Written informed consent was acquired from participants before being enrolled in INTERMACS. The Data Coordinating Center at University of Alabama at Birmingham provided us the data once it was de-identified. The data used in the present study was anonymized and de-identified prior to analysis. Inclusion criteria for this study was: use of a continuous flow LVAD as the primary implant and age>19, thus excluding pediatric patients. Patients who ultimately received an Right Ventricular Assist Device (RVAD) were included as long as the initial implant was an LVAD and an RVAD was placed thereafter. The specific type of RVAD was not considered. Total Artificial Heart recipients were excluded from this study. This translated to 8,050 patients from year 2006 to 2013 in the initial dataset. Data from patients whose LVAD was electively removed (e.g., due to transplantation or recovery) were censored at the time of event (See Table 1).

**Table 1:** Mortality statistics, censored for explant and transplant.

Endpoint	Survival No. (%)	Death No. (%)	Total	Survival SMOTE No. (%)	Death SMOTE No. (%)	Total SMOTE No. (%)
30 Day	7620 (95.2)	387 (4.8)	8007	7620 (90.8)	774 (9.2)	8394
90 Day	7024 (90.5)	737 (9.5)	7761	7024 (82.6)	1474 (17.3)	8498
6 Month	6245 (86.1)	1007 (13.9)	7252	6245 (75.6)	2014 (24.4)	8259
1 Year	5241 (79.7)	1334 (20.3)	6575	5241 (66.2)	2668 (33.7)	7909
2 Year	4432 (72.7)	1667 (27.3)	6099	4432 (57.1)	3334 (42.9)	7766

SMOTE: synthetic minority oversampling technique.

### Pre-processing

The raw data from the registry was pre-processed to transform continuous data into discrete bins (required by the Bayesian algorithm) and to fill in missing data, described below.

#### Discretization

Discretization was done based on a balance of equal frequency binning and published upper and lower limits of clinically normal values [13], [14]. Table S1 provides the list of variables with their respective formats and input values. Clinical scenarios and outcomes, such as events during hospitalization, adverse events prior to implant and interventions within 48 hours of implant, were defined using the INTERMACS definitions.

#### Missing data

The patient records provided by the INTERMACS data set were found to be routinely incomplete (see Table S1 for percentages missing). Missing data was separated into two categories: *missing at random* (MAR), and *missing not at random* (MNAR) [15]. Missing demographics and lab values, for example, were considered to be either MAR, in which case we assumed the most probable values (i.e. BMI between 24–27 since it is considered normal). The method of using normal values has been cited in previous studies to produce superior results compared to listwise deletion and other methods [16]. Missing co-morbidities, on the other hand were considered MNAR, in which case we assumed that it was not a concern for that patient. This is standard procedure for handling MNAR data in medical datasets. For example, if a patient has no record of a chest x-ray then the doctor probably did not feel the need to order one. In these cases, it is common to assume a “normal” value, which in the case of the x-ray would be *not ordered* as opposed to *unknown*, or simply *missing*. Additional data in the MAR category were missing laboratory and hemodynamic values were designated as *not ordered*. Missing medication data was considered MNAR, in which we assumed that no such medication was prescribed. Finally, missing quality of life metrics were considered MNAR, and designated as *unknown*. This was justified based on voluntary and sporadic participation in the two quality of life surveys, the EuroQoL [17] (offered since the beginning of the registry)and the Kansas City Cardiomyopathy Questionnaire (KCCQ) [18] implemented after 2012.

### Synthetic Minority Oversampling Technique (SMOTE)

A common challenge in data mining that inhibits the predictive ability of the model, is an uneven distribution of the outcome (survival post-LVAD). In medicine, there is often a larger portion of the cohort that are free of death or adverse event as compared to the number of events. The 30-day mortality rate in INTERMACS is 4.8%, compared to a 95.2% survival rate. With such a discrepancy, the model will always predict the majority class (survival) if uncertain how to classify a new patient. This will pose a setback clinically, as it is most important to identify the high-risk patients (early mortality).

One of the most frequently cited methods for addressing this bias is SMOTE [19], which is used when the minority class is increased by creating synthetic examples rather than by over-sampling with replacement. The minority class (mortality) is over-sampled by taking each minority class sample and introducing synthetic examples along the line segments joining any or all of the k minority class nearest neighbors. For this study we increased the minority class by 100%, which would essentially double the mortality number at each endpoint, while the survival number remained unchanged. The 100% was identified by incrementally increasing the percentage from 0% to 100% and identifying the best performing percentage. We set the cut off at 100% to ensure there would be at least an even number of actual and synthetic cases, as opposed to more synthetic compared to actual cases. Synthetic samples are generated by taking the difference between the instance (or patient) under consideration and its nearest neighbor. This difference was multiplied by a random number between 0 and 1, and added to the feature vector under consideration. (This causes the selection of a random point along the line segment between two specific features.) SMOTE effectively forces the decision region of the minority class to become more general. Table 1 juxtaposes the dataset before and after application of SMOTE.

### Bayesian Networks

The machine learning methods used for the present study were built upon Bayesian techniques used previously by our group for multiple decision support studies, including: optimal VAD weaning [20], the need for right ventricular support due to right ventricular failure [21]–[23] and a two-center study to predict 90-day survival for continuous flow LVADs [24], [25]. Bayesian networks (BNs) [26] are acyclic directed graphs in which nodes represent random variables and directed arcs (represented as arrows) between pairs of variables represent influences between them. In addition to the graph structure, a BN is equipped with conditional probability tables (CPT), associated with each node, and describes the probability distribution over the variable's values conditional on all combinations of values of its immediate predecessors (parents) in the graph. A BN is a representation of a factorization of the joint probability distribution over its variables. Independence between a pair of variables is represented by absence of a directed arrow between these variables. Explicit representation of independences results in significant savings in the number of parameters necessary to represent the complete joint probability distribution, making BNs highly practical even in very complex domains.

The joint probability distribution represented by a BN can be updated in the light of new evidence by means of Bayes theorem. Efficient algorithms exist that given observed values of some of the variables, produce the new joint (conditional) probability distribution over the remaining variables. While the quality of the results rests on the quality of the underlying representation of the joint probability distribution, BNs have been shown to be very robust to precision of their parameters [27] and there are indications that possible errors in structure (i.e., incorrect independences) have also limited influence on the quality of the results [28].

To illustrate, one may consider a simple BN model in Figure S1, modeling risk factors related to LVAD survival. The network models the joint probability distribution over the four factors and the survival variable. Not all variables need to be known. For example, we could derive from the network the average survival probability for a center with limited LVAD experience.

For this study, we evaluated four BNs: *Näıve Bayes*, *Tree-Augmented Näıve Bayes*, *Bayesian Search* and the *Greedy Thick Thinning Algorithm* [29]. We ultimately chose the *Greedy Thick Thinning Algorithm* as the final model based on a tradeoff between complexity and accuracy. The model starts with an empty graph and iteratively adds and removes arcs as it builds the network, incrementally increasing the Bayesian scoring metric: in this case, the K2 prior distribution over the parameters [30].

### Markov Blanket

We applied the Markov Blanket to simplify the network and reduce the likelihood of over-fitting the model to the dataset (i.e. performs well only on the training data, but unable to generalize to other datasets). It has been shown in previous studies that using the Markov Blanket is one of the most effective and efficient methods of feature selection [31]. The Markov Blanket for each of the BN models was comprised of the class node and the family of nodes that make it conditionally independent of all other nodes in the network. This includes the parents, the children and the parents of the children, or spouses [26].

### Evaluation

Ten-fold cross validation was the vehicle for model derivation and optimization. The BN classifiers were evaluated on an independent testing/holdout dataset comprised of a training set of approximately 90% of the data records and a testing set from the remaining approximate 10%. The models were derived, built and implemented using two open-source machine learning software libraries: The GeNIe modeling environment developed by the Decision Systems Laboratory of the University of Pittsburgh [32] and the machine learning library WEKA (Waikato Environment for Knowledge Analysis) [33]. Performance metrics included: Accuracy, True Positive, True Negative and area under the ROC curve (AUC). A sensitivity analysis was also performed, where all variables are kept constant and a single parameter is changed to observe the direct affects. This is then expanded to change additional parameters to visualize their additive affects.

## Results

**Figure 1:**
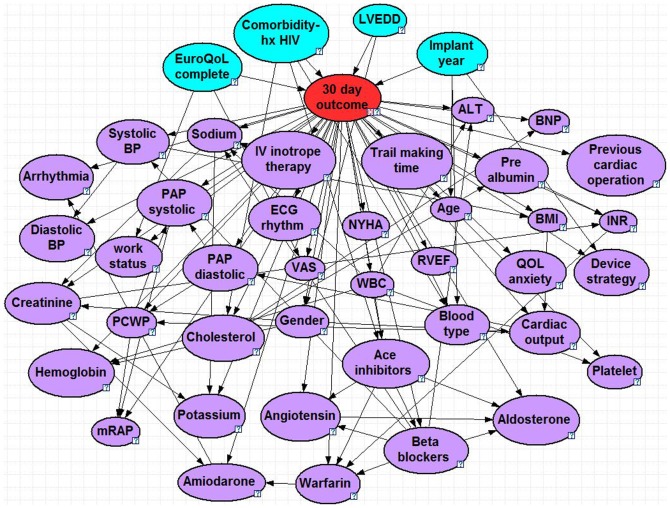
30 Day Bayesian Model. Node colors: red =  class, blue =  parent, purple =  child. Question marks identify nodes that do not have specific evidence set and use the population distribution as the prior distribution. LVEDD =  left ventricle end diastolic diameter, ALT =  alanine transaminase, BP =  blood pressure, mRAP =  mean right atrial pressure, PCWP =  pulmonary capillary wedge pressure, VAS =  visual analog scale, BNP =  B-type natriuretic peptide, WBC =  white blood cell, NYHA =  New York Heart Association functional class, RVEF =  right ventricle ejection fraction, INR =  international normalized ratio, BMI =  body mass index, ECG =  Electrocardiography, QOL =  quality of life, hx HIV =  history of human immunodeficiency virus.

**Figure 2:**
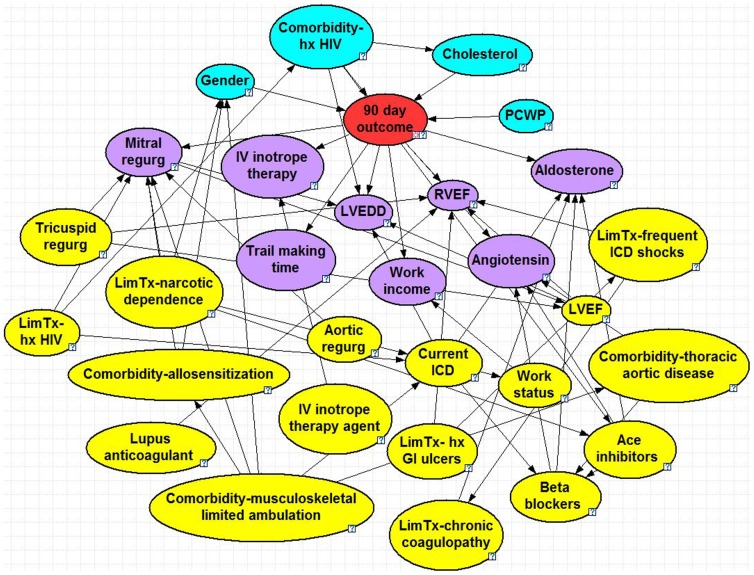
90 Day Bayesian Model. Node colors: red =  class, blue =  parent, purple =  child, yellow =  spouse. Question marks identify nodes that do not have specific evidence set and use the population distribution as the prior distribution. LVEDD =  left ventricle end diastolic diameter, PCWP =  pulmonary capillary wedge pressure, RVEF =  right ventricle ejection fraction, LVEF =  left ventricle ejection fraction, hx HIV =  history of human immunodeficiency virus, ICD =  implantable cardioverter defibrillator, Lim tx =  limitation for transplant listing, GI =  gastrointestinal, IV =  intravenous.

**Figure 3:**
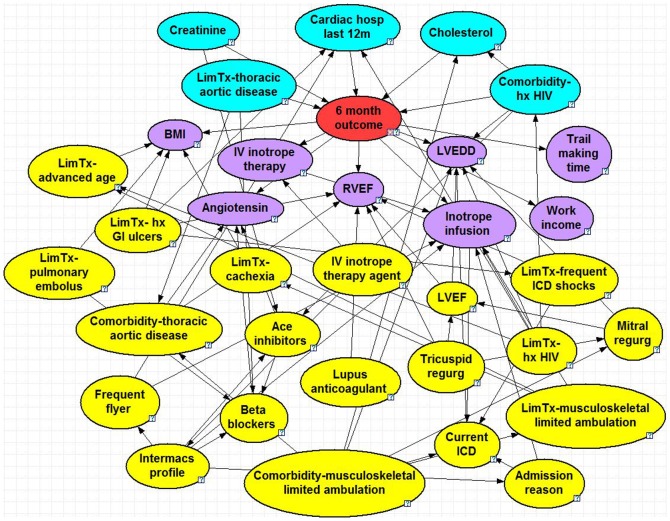
6 Month Bayesian Model. Node colors: red =  class, blue =  parent, purple =  child, yellow =  spouse. Question marks identify nodes that do not have specific evidence set and use the population distribution as the prior distribution. LVEDD =  left ventricle end diastolic diameter, RVEF =  right ventricle ejection fraction, LVEF =  left ventricle ejection fraction, hx HIV =  history of human immunodeficiency virus, BMI =  body mass index, ICD =  implantable cardioverter defibrillator, Lim tx =  limitation for transplant listing, GI =  gastrointestinal, IV =  intravenous.

**Figure 4:**
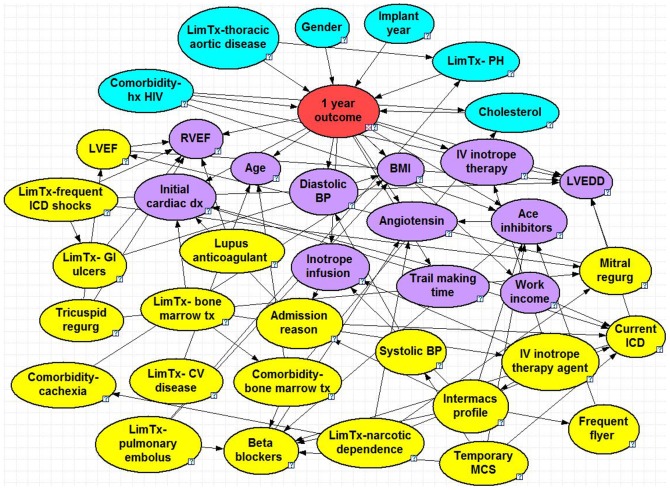
1 Year Bayesian Model. Node colors: red =  class, blue =  parent, purple =  child, yellow =  spouse. Question marks identify nodes that do not have specific evidence set and use the population distribution as the prior distribution. LVEDD =  left ventricle end diastolic diameter, RVEF =  right ventricle ejection fraction, BMI =  body mass index, lim tx PH =  limitation for transplant listing due to pulmonary hypertension, IV =  intravenous, ICD =  implantable cardioverter defibrillator, CV =  cardiovascular, GI =  gastrointestinal, BP =  blood pressure, hx HIV =  history of human immunodeficiency virus, MCS =  mechanical circulatory support.

**Figure 5:**
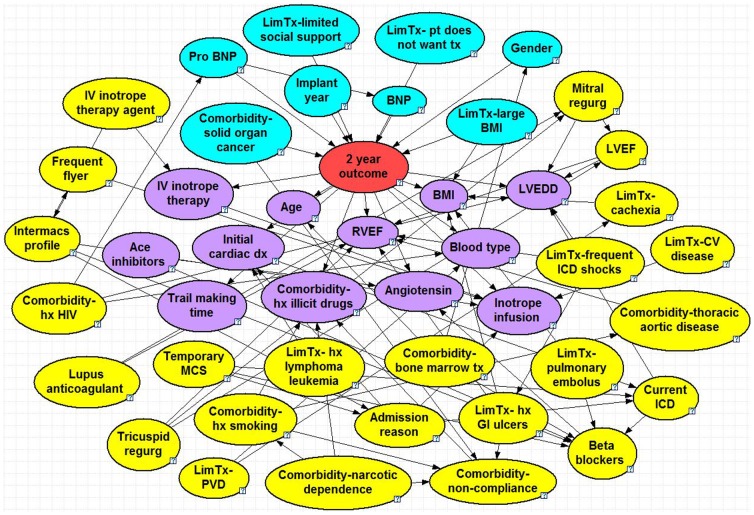
2 Year Bayesian Model. Node colors: red =  class, blue =  parent, purple =  child, yellow =  spouse. Question marks identify nodes that do not have specific evidence set and use the population distribution as the prior distribution. LVEDD =  left ventricle end diastolic diameter, BNP =  B-type natriuretic peptide, LVEF =  left ventricle ejection fraction, GI =  gastrointestinal, IV =  intravenous, ICD =  implantable cardioverter defibrillator, PVD =  peripheral vascular disease, hx HIV =  history of human immunodeficiency virus, BMI =  body mass index, MCS =  mechanical circulatory support, CV =  cardiovascular.

The optimized BNs can be seen in Figures 1–5, and a summary of their performance can be found in Table 2. Accuracy was greatest for the 30 day model with 95% and lowest for the 2 year model with 71%. The True Positive (proportion of patients who were correctly predicted to not survive) was greatest for the 2 year at 65% and lowest for the 90 day model at 23%. The True Negative (proportion of patients who were correctly predicted to survive past the endpoint) was greatest for the 30 day model at nearly 100% and lowest for the 2 year model at 76%. The ROC % was greatest for the 30 day model at 93% and lowest for the 6 month, 1 year and 2 year models (all 71%).

**Table 2:** Summary of Bayesian Model Performance.

Endpoint	Accuracy (%)	True Positive (Dead) (%)	True Negative (Alive) (%)	ROC (%)
30 Day	94.9	51.0	99.4	92.5
90 Day	84.2	22.9	97.1	73.9
6 Month	78.2	30.3	93.7	70.6
1 Year	73.1	49.1	85.3	70.6
2 Year	71.4	65.0	76.2	70.8

ROC =  receiver operating characteristic curve.

### 30 Day Model

The 30-day mortality model consisted of 44 nodes and 93 arcs. There are four parent nodes for 30 day mortality: whether or not a patient completed the EuroQoL survey, a history of HIV, implant year, and left ventricular end diastolic dimension (LVEDD), an indicator of dilated cardiomyopathy. Positive HIV was the strongest predictive node, followed by the implant year. When a patient is indicated to have a history of HIV, then the baseline likelihood of survival drops from 95% (when no patient-specific evidence is specified) to 53% and drops further to 43% chance of survival when the LVEDD is above 75 mm. The children nodes includes a combination of laboratory values (sodium, BNP and platelets), hemodynamics (mean right arterial pressure, diastolic blood pressure and ECG rhythm), demographics (age and gender) and pre-implant medication (beta blockers, aldosterone and amiodarone). This model was the one exception to using the Markov Blanket, as it would approach nearly 150 nodes with the inclusion of the spousal nodes and would be more prone to over-fitting the dataset. For the model, we simply used the parents and children nodes, which put it at a comparable size as the other endpoint Bayesian models.

### 90 Day Model

The 90-day model consisted of 30 nodes and 56 arcs. There are four parent nodes: gender, co-morbidity of HIV, cholesterol and pulmonary capillary wedge pressure. History of HIV decreased 90-day chance of survival from 87% to 52% and, when combined with elevated cholesterol (above 150 mg/dL), further decreases to 47%. The chance of survival increases (as compared to the baseline) to 91% if a patient's cholesterol is below 110 mg/dL and they do not have HIV. The children nodes include variables such as mitral regurgitation, right ventricular ejection fraction and work income. Spouse nodes include tricuspid and aortic regurgitation, pre-implant beta blockers, pre-implant ace inhibitors, and left ventricular ejection fraction.

### 6 Month Model

The 6-month model consisted of 34 nodes and 70 arcs. There are five parent nodes: limitation for transplant due to thoracic aortic disease, creatinine, number of cardiac hospitalizations within the 12 months prior to LVAD implant, cholesterol and HIV. Compared to the baseline chance of survival at 6 months of 76%, the limitation for transplant reduces to 48%, and when combined with creatinine levels below 1.1 mg/dL further reduces to 37%. The children nodes include variables such as BMI, work income and angiotensin. Spouse nodes include presence of an implantable cardioverter defibrillator (ICD), admission reason before implant, history of gastrointestinal bleeding, limitation for transplant listing due to advanced age, and the INTERMACS profile (from level 1 critical cardiogenic shock to level 7 resting heart failure symptoms but stable).

### 1 Year Model

The 1-year model consisted of 39 nodes and 76 arcs. There are six parent nodes: co-morbidity of HIV, limitation for transplant listing due to thoracic aortic disease, gender, implant year, limitation for transplant listing due to pulmonary hypertension and cholesterol. The baseline chance of survival at 1 year post-implant is 75%, and more recent implants (2013) have higher chances of survival (86%) compared to implants done between 2008–2010 (64–66%). Although this is likely due to shorter follow up times for the more recent implants, it may also reflect advances in implant techniques and changes in the patient post-VAD management. Similar to the other models, both a history of HIV and thoracic aortic disease have major impacts on the chance of 1 year survival. The children nodes include age, right ventricular ejection fraction, BMI, ace inhibitors and diastolic blood pressure. Spouse nodes include admission reason, systolic blood pressure, the INTERMACS profile, temporary mechanical circulatory device, current ICD and co-morbidity due to cachexia (malnutrition).

### 2 Year Model

The model for 2-year mortality consisted of 45 nodes and 78 arcs. There are eight parent nodes: pro-BNP, BNP, co-morbidity of solid organ cancer, implant year, gender, limitation for transplant listing due to limited social support, patient refusal for transplant listing, and limitation for transplant listing due to large BMI. The baseline 2-year chance of survival is 69%, which increases by 5% if the patient's BNP levels are below 540 pg/dL and increases further to 84% if the implant was done more recently. The chance of survival falls below the baseline (to 62%) if BNP is elevated above 1200 pg/dL, drops further to 58% if the patient declines transplant listing and drops even further to 50% if the patient also has limited social support. The children nodes included age, trail making time (a neuro-psychological test of visual attention and task switching), co-morbidity due to a history of illicit drug use and blood type. Spousal nodes included limitation for transplant listing due to peripheral vascular disease, the INTERMACS profile, being a so-called *frequent flyer* (patients who are repeatedly in and out of the hospital emergency room), the IV inotrope therapy agent and age.

### HMRS

The HMRS was derived and validated for 90-day and 1-year mortality and identifies patients as either low risk (4–8% mortality), medium risk (11–16% mortality) or high risk (25–29% mortality). When applied to the INTERMACS database, 93.1% of patients were predicted as low risk with a 9% morality rate, 4.4% were predicted as medium risk with a 16% mortality rate, and 2.3% were predicted as high risk with a 14.6% mortality rate. The ROC for the 90-day HMRS score was 60.3% compared to 73.9% for the 90-day CHRiSS model and the 1-year HMRS score 57.4% compred to 70.9% for the 1-year CHRiSS model.

## Discussion

The decision to refer a patient for LVAD therapy entails processing numerous, dynamically evolving and inter-related clinical variables, which is a complex task filled with uncertainty. The trajectory by which a patient may be offered the option to receive a LVAD involves multiple specialties and decision points. The extensive volume of information and data that must be considered (demographics, labs, history, family support, insurance, etc.) is at odds with the rapidity with which some decisions must be made. Deferring a decision to implant an LVAD, may allow the severity of ESHF to progress thereby increasing the likelihood of an adverse event. Lietz and Miller describe an “optimal window” for LVAD implantation, beyond which the operative risk may deem the intervention futile. However, the key to successful and timely implementation of LVAD therapy is the proper identification of the patient who will benefit from this type of therapy: before clinical instability occurs, which has a major impact on the downstream morbidity and mortality with this intervention.

The advanced heart failure team must evaluate risk in terms of both immediate complications as well as likelihood of repeated readmissions due to longer-term complications. Hence there is a need for a CDSS to aid the physicians in pre-LVAD candidate assessment, by providing personalized risk predictions to ultimately encourage earlier intervention in the appropriate patients. We envision two steps (with iteration) when using CHRiSS: the clinician would first input the variables available for that patient and calculate their initial risk and then adjust actionable variables to perform the “what if” scenarios. For example, a clinician assessing a patient who is initially malnourished (has lower levels of albumin), could then adjust the albumin to the normal range and see if the prognosis improves and by how much.

Such a dynamic CDSS does not exist for advanced HF patients and the BNs offer several advantages over the traditional statistical methods used to derive risk scores such as the HMRS or DTRS. Current risk scores are limited by their: (1) simplistic four to nine variable summed scoring system; (2) inability to co-evolve with the changing HF risk factors and emerging treatment options; and (3) requirement to know a fixed set of variables and inability to be computed when any are missing. BNs address these limitations, as they are able to: (1) account for hundreds of inter-related variables in a single model; (2) dynamically update as risk factors change and different drugs are created; and (3) compute predictions with missing values by using the prior probabilities encoded within each variable node. The current study demonstrated the potential for developing advanced CDSS based on these models in the domain of HF, as well as other medical applications.

For example, in the absence of any decision tool, expert judgment resulted in the correct prognosis of survival at 1 year 79.7% (n = 5241 patients) of the time (true survival rate, see Table 1). This translates to 20.3% (n = 1334 patients) frequency of incorrect judgment of survival. (Presuming that the decision to implant an LVAD in these patients was predicated on positive 1-year survival prognosis.) With the added contribution of CHRiSS in decision-making, 85.3% (see True Negative in Table 2) of these 79.7% (actual survival from Table 1) would be corroborated by the prognostic model. Thereby denying an LVAD from 14.7% of these patients who would have survived, had the physician followed CHRiSS recommendation. On the other hand, the expert incorrectly predicted survival in 20.3% (n = 1334, mortality % from Table 1). CHRiSS would have predicted almost half of these patients, hence sparing 667 patients from a suboptimal outcome. Although there is no way of knowing, retrospectively, how long these LVAD patients would have survived if they did not receive a LVAD, the additional insight provided by CHRiSS would assist the patient and physician in weighing the risks and benefits of the various courses of treatment. For example, these patients may be prioritized for cardiac transplantation, thereby making better use of limited organ supply for those who would not otherwise survive on a LVAD (in exchange for a transplant patient who would have done just as well on a LVAD.)

The 2-year CHRiSS model correctly identified 65% of patients who would not survive past the endpoint, which would be very important to know for DT patients to decide if they wish to accept this risk considering chance of surviving (and thriving) for many years with this end of life treatment. Following the same logic for predicting short-term outcomes, CHRiSS would spare half of the 387 patients from undergoing a major surgery who would do very poorly during the recovery post-implant. The 30-day model could aid in vetting potential high risk DT and bridge to transplant (BTT) candidates. Paradoxically, the performance of the 90-day and 6-month models were not as good as the 30-day, 1-year, and 2-year models. This may be attributable to the heterogeneity in causes of death, which may confound the Bayesian Network to identify specific predictive variables.

We had also conducted a sensitivity analysis to observe the direct effects of each variable on the outcome, as well as the additive effects of several variables. The variables that created the greatest effect on the outcome were presented in the results section for each model. For the short-term outcome, positive HIV increased likelihood of mortality by 42% at 30-days and 35% at 90-days. Each of these percentages were further increase by 5–10% when adding an enlarged LVEDD (dilated cardiomyopathy) or elevated cholesterol, respectively. When considering long-term predictors (2-year model), one of the parent nodes was BNP, which had bi-modal effects: normal levels lead to reduced chance of mortality by 5% and elevated levels increased mortality by 7%.

The use and acceptance of Bayesian methodology are becoming increasingly prevalent in the medical community (see, for example [34]–[42]). For example, in 2010, the FDA released a guidance for the use of Bayesian statistics in medical device clinical trials [43]. In 2013, the United Network for Organ Sharing (UNOS) proposed the adoption of a new Bayesian methodology to better identify those transplant programs that may be underperforming in the area of patient and graft survival.

Although the methods described in this study are more advanced than the current risk scores used in HF prognosis, the results are far from perfect. There are several limitations that may adversely affect performance, including inherent retrospective bias, imbalance of the outcome variable, and extensive missing data. We aim to address these limitations in our ongoing prospective studies. It is also important to clarify that the CHRiSS models are able to discern likelihood of mortality for patients receiving an LVAD and does not allude to any predictions for patients not receiving the device. We plan to perform a follow up study that will derive BN models based on both INTERMACS data, as well as a HF cohort for those who do not receive an LVAD. Nevertheless, the current models are able to provide decision support to the HF clinicians regarding potential future LVAD recipients.

This study was the first application of the Bayesian Network algorithm to a cohort of LVAD recipients. Although the current models outperform the current LVAD risk scores, there is an opportunity to improve them yet further as additional prospective data is collected, and additional risk factors are added to the model. Most certainly, the Bayesian models better represent the complex inter-variable relationships between clinical variables, better emulating human logic, which in turn makes them more appealing to end users.

## Supporting Information

Figure S1 **A simple Bayesian network model modeling risk factors related to LVAD survival.**
(TIFF)

Table S1 **Many =  not all format options listed, DT =  destination therapy, BTT =  bridge to transplant, BTR =  bridge to recovery, CPB =  cardiopulmonary bypass, MI =  myocardial infarction, ECMO =  extracorporeal membrane oxygenator, CABG =  coronary artery bypass graft, IABP =  intra-aortic balloon pump, INR =  international normalized ratio, ICD =  implantable cardioverter defibrillator, BNP =  B-type natriuretic peptide, WBC =  white blood cell, ALT =  alanine transaminase, AST =  aspartate aminotransferase, CRP =  C-Reactive Protein, LVEF =  left ventricle ejection fraction, LVEDD =  left ventricle end diastolic diameter, RVEF =  right ventricle ejection fraction, PCWP =  pulmonary capillary wedge pressure, NYHA =  New York Heart Association functional class, HF =  heart failure, GI =  gastrointestinal, HIV =  human immunodeficiency virus.**
(DOCX)
