# Supplementary figures and images for: Cardiac Health Risk Stratification System (CHRiSS): A Bayesian-Based Decision Support System for Left Ventricular Assist Device (LVAD) Therapy
Source: PLoS One. 2014 Nov 14;9(11):e111264. doi: 10.1371/journal.pone.0111264 (PMC4232308; doi:10.1371/journal.pone.0111264)

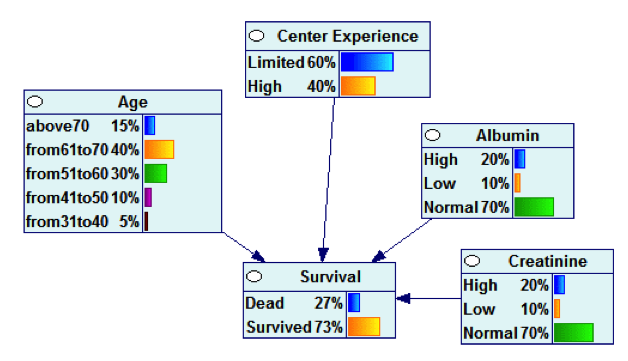

Supplement: Figure S1 — A simple Bayesian network model modeling risk factors related to LVAD survival. (TIFF) [file pone.0111264.s001.tiff]
