# Supplementary material for: Cardiac Health Risk Stratification System (CHRiSS): A Bayesian-Based Decision Support System for Left Ventricular Assist Device (LVAD) Therapy
Source: PLoS One. 2014 Nov 14;9(11):e111264. doi: 10.1371/journal.pone.0111264 (PMC4232308; doi:10.1371/journal.pone.0111264)
Supplement: Table S1 — Many = not all format options listed, DT = destination therapy, BTT = bridge to transplant, BTR = bridge to recovery, CPB = cardiopulmonary bypass, MI = myocardial infarction, ECMO = extracorporeal membrane oxygenator, CABG = coronary artery bypass graft, IABP = intra-aortic balloon pump, INR = international normalized ratio, ICD = implantable cardioverter defibrillator, BNP = B-type natriuretic peptide, WBC = white blood cell, ALT = alanine transaminase, AST = aspartate aminotransferase, CRP = C-Reactive Protein, LVEF = left ventricle ejection fraction, LVEDD = left ventricle end diastolic diameter, RVEF = right ventricle ejection fraction, PCWP = pulmonary capillary wedge pressure, NYHA = New York Heart Association functional class, HF = heart failure, GI = gastrointestinal, HIV = human immunodeficiency virus. (DOCX) [file pone.0111264.s002.docx]

| **Supplemental Table 1: Pre-implant variables from INTERMACS (n=221)** | | |
| --- | --- | --- |
| **Demographics (n=32)** | **% missing** | **Levels** |
| Age interval | 0 | 19-29, 30-39, 40-49, 50-59, 60-69, 70-79, 80+ |
| Gender | 0 | male/female |
| Quarter of Implant | 0 | Q1, Q2, Q3, Q4 |
| Implant year | 0 | 2006, 2007,…2013 |
| BMI | 1 | < 24, 24-27, 27-32, > 32 |
| Education level | 23 | Many: Grade school, High school, Bachelors… |
| Work status | 9 | Many: full time, disability, part time… |
| Work income | 7 | yes/no |
| NYHA | 9 | Class I, II, III, IV |
| INTERMACS profile | 0 | Profile 1-7 |
| Device strategy | 0 | Many: DT, BTT, BTR |
| VAD indication | 0 | None, Failure to wean from CPB, post cardiac surgery |
| Admission reason | 0 | Many: Acute MI, Heart Failure, Cardiac surgery… |
| Time since first cardiac diagnosis | 4 | < 1 month, 1 month-1year, 1-2 years, >2 years |
| Primary diagnosis | 1 | Many: Dilated myopathy, coronary artery disease, valvular heart disease… |
| Known cardiac biopsy | 0 | Many: no biopsy known, Sarcoidosis, myocarditis… |
| Previous cardiac operation | 0 | Many: ECMO, CABG, valve replacement, multiple… |
| Current ICD | 1 | yes/no |
| Events this hospitalization | 1 | Many: MI, intubation, dialysis… |
| Intervention within the last 48 hours | 0 | Many: IABP, ECMO, feeding tube… |
| Temporary circulatory support | 27 | yes/no |
| VAD related study | 76 | yes/no |
| Primary diagnosis- congenital | 99 | Many: transposition of the great arteries, atrial or ventricular septal defect, not congenital |
| Previous cardiac operation- congenital surgery | 99 | Many: VSD or ASD repair, corrected transposition, not congenital |
| Infection type | 94 | Bacterial, fungal, viral, no infection |
| Infection location | 94 | Pneumonia, blood, endocarditis, urinal, mediastinum, no infection |
| Number of cardiac hospitalizations in last 12 months | 69 | 0-1, 2-3, 4 or more |
| Frequent flyer | 76 | yes/no |
| Cardiac resynchronization therapy | 64 | yes/no |
| **Co-morbidities (Limitation for transplant listing) (n=82)** |  |  |
| Advanced age (limitation tx) | 8 (68) | yes/no |
| Frailty (limitation tx) | 8 (68) | yes/no |
| Patient does not want transplant (limitation tx) | 8 (68) | yes/no |
| Musculoskeletal limitation for ambulation (limitation tx) | 8 (68) | yes/no |
| Immunosuppression (limitation tx) | 8 (68) | yes/no |
| Allosensitization (limitation tx) | 8 (68) | yes/no |
| Chronic renal disease (limitation tx) | 8 (68) | yes/no |
| Recent pulmonary embolus (limitation tx) | 8 (68) | yes/no |
| Thoracic aortic disease (limitation tx) | 8 (68) | yes/no |
| Large BMI (limitation tx) | 8 (68) | yes/no |
| Malnutrition cachexia (limitation tx) | 8 (68) | yes/no |
| Heparin induced thrombocytopenia (limitation tx) | 8 (68) | yes/no |
| Chronic infectious concerns (limitation tx) | 8 (68) | yes/no |
| Limited cognition understanding (limitation tx) | 8 (68) | yes/no |
| Limited social support (limitation tx) | 8 (68) | yes/no |
| Repeated non-compliance (limitation tx) | 8 (68) | yes/no |
| History of illicit drug use (limitation tx) | 8 (68) | yes/no |
| Severe depression (limitation tx) | 8 (68) | yes/no |
| Other major psychiatric diagnosis (limitation tx) | 8 (68) | yes/no |
| Other comorbidities (limitation tx) | 8 (68) | yes/no |
| Pulmonary disease (limitation tx) | 8 (68) | yes/no |
| Pulmonary hypertension (limitation tx) | 8 (68) | yes/no |
| Unfavorable mediastinal anatomy (limitation tx) | 8 (68) | yes/no |
| Severe diabetes (limitation tx) | 8 (68) | yes/no |
| Major stroke (limitation tx) | 8 (68) | yes/no |
| Peripheral vascular disease (limitation tx) | 8 (68) | yes/no |
| History of solid organ cancer (limitation tx) | 8 (68) | yes/no |
| History of lymphoma leukemia (limitation tx) | 8 (68) | yes/no |
| History of alcohol abuse (limitation tx) | 8 (68) | yes/no |
| Currently smoking (limitation tx) | 8 (68) | yes/no |
| Frequent ICD shock (limitation for tx) | 68 (68) | yes/no |
| History of atrial arrhythmia | 68 (68) | yes/no |
| History of GI ulcers | 68 (68) | yes/no |
| History of liver dysfunction | 68 (68) | yes/no |
| History of chronic coagulopathy | 68 (68) | yes/no |
| History of bone marrow transplant | 68 (68) | yes/no |
| History of HIV | 68 (68) | yes/no |
| History of narcotic dependence | 68 (68) | yes/no |
| History of hepatitis | 68 (68) | yes/no |
| Other cardiovascular disease | 68 (68) | yes/no |
| History of smoking | 68 (68) | yes/no |
| **Laboratory (n=20)** |  |  |
| Blood type | 2 | A, B, O, AB |
| Creatinine | 0 | not done, <1.1, 1.1-1.5, >1.5 |
| Sodium | 0 | not done, <133, 133-137, >137 |
| INR | 1 | not done, <1.2, 1.2-1.5, >1.5 |
| Albumin | 1 | not done, <3.2, 3.2-3.8, >3.8 |
| Pre albumin | 3 | not done, <15, 15-21, >21 |
| BNP | 4 | not done, <540, 540-1200, >1200 |
| Pro BNP | 4 | not done, <3000, 3000-7000, >7000 |
| WBC | 0 | not done, <7, 7-10, >10 |
| Platelet | 0 | not done, <150, 150-220, >220 |
| AST | 1 | not done, <25, 25-40, >40 |
| ALT | 1 | not done, <20, 20-40, >40 |
| Cholesterol | 4 | not done, <110, 110-150, >150 |
| Potassium | 0 | not done, <3.9, 3.9-4.3, >4.3 |
| Hemoglobin | 0 | not done, <10, 10-12.5, >12.5 |
| CRP | 4 | not done, <3, 3-10, >10 |
| Uric acid | 62 | not done, <7, 7-10, >10 |
| Lymphocyte count | 62 | not done, <12, 12-20, >20 |
| Lactate dehydrogenase | 60 | not done, <225, 225-330, >330 |
| Lupus anticoagulant | 98 | positive/negative |
| **Hemodynamics (n=24)** |  |  |
| LVEF | 9 | not recorded, <20 (severe), 20-29 (moderate/severe), 30-39 (moderate), 40-49 (mild), >50 (normal) |
| LVEDD | 13 | not done, N/A, <6.5, 6.5-7.5, >7.5 |
| RVEF | 23 | not done, mild, normal, moderate, severe |
| Pulmonary artery systolic pressure | 23 | not done, <45, 45-60, >60 |
| Pulmonary artery diastolic pressure | 23 | not done, <20, 20-30, >30 |
| Mean right atrial pressure | 27 | not done, <10, 10-15, >15 |
| PCWP | 27 | not done, <20, 20-30, >30 |
| Heart rate | 1 | not done, <80, 80-95, >95 |
| Systolic blood pressure | 1 | not done, <100, 100-120, >120 |
| Cardiac output | 35 | not done, <3.5, 3.5-5, >5 |
| Diastolic blood pressure | 1 | not done, <60, 60-70, >70 |
| ECG rhythm | 3 | Many: sinus, paced, Afib… |
| 6 minute walk | 5 | not done, <465, 465-750, 750-1030, >1030 |
| VO_2_ max | 10 | not done, <10, 10-13, >13 |
| Peak R | 14 | not done, <1, 1, 1.1, >1.2 |
| Mitral regurgitation | 9 | not done, none, mild, moderate, severe |
| Tricuspid regurgitation | 10 | not done, none, mild, moderate, severe |
| Aortic regurgitation | 11 | not done, none, mild, moderate, severe |
| Ascites | 10 | yes/no |
| Peripheral edema recorded | 6 | yes/no |
| Arrhythmia | 5 | yes/no |
| Central venous pressure | 65 | not done, <8, 8-13, >13 |
| Cardiac index | 62 | not done, <2, 2-2.5, >2.5 |
| Doppler opening pressure | 66 | not done, <65, 65-75, 75-85, >85 |
| **Medication (n=19)** |  |  |
| Angiotensin | 9 | no, known previous use (within past year), currently using |
| Amiodarone | 5 | no, known previous use (within past year), currently using |
| Ace inhibitors | 6 | no, known previous use (within past year), currently using |
| Beta blockers | 3 | no, known previous use (within past year), currently using |
| Aldosterone | 6 | no, known previous use (within past year), currently using |
| Loop diuretics | 0 | yes/no |
| Warfarin | 5 | no, known previous use (within past year), currently using |
| Antiplatelet | 5 | no, known previous use (within past year), currently using |
| Neseritide | 1 | yes/no |
| Nitric oxide | 1 | yes/no |
| Inotrope infusion | 2 | yes/no |
| IV inotrope therapy | 1 | yes/no |
| IV inotrope therapy agent | 20 | Many: Milrinone, Dopamine, Dobutamine… |
| Allopurinol | 63 | no, known previous use (within past year), currently using |
| Metalozone | 61 | no, known previous use (within past year), currently using |
| Metalozone frequency | 96 | none, intermittent, regular |
| Phosphodiesterase | 61 | no, known previous use (within past year), currently using |
| Diuretic type | 68 | Bumetanide, Furosemide, Torsemide, multiple, none |
| Diuretic dose | 68 | not taking, <20, 20-60, 60-120, >120 |
| **Quality of Life (n=44)** |  |  |
| Completed EuroQoL | 3 | yes, N/A |
| EurQoL main occupation | 46 | Many: retired, student, working … |
| EuroQoL mobility | 45 | unknown, no problem, some problems, major problems |
| EuroQoL self-care | 45 | unknown, no problem, some problems, major problems |
| EuroQoL activities | 45 | unknown, no problem, some problems, major problems |
| EuroQoL pain | 45 | none, moderate, extreme |
| EuroQoL anxiety | 45 | none, moderate, extreme |
| Visual Analog Status health state | 46 | <25, 25-40, 40-65, >65 |
| Currently smoke cigarettes | 46 | yes/no |
| Trail making status | 0 | completed, attempted but not completed, no attempted, completed but invalid |
| Reason EuroQoL not completed | 66 | Many: too sick/tired, no time, can’t concentrate, too stressed, N/A (completed) |
| Close friends seen per month | 82 | unknown, <6, 6-10, 10-20, >20 |
| Lost 10 lbs in last year | 80 | unknown, yes, no |
| Cigarettes smoked (average) | 99 | unknown, <half pack, half-1 pack, 1-2 packs, >2 packs |
| Coping with health related stress | 81 | unknown, stress level 1-10 |
| VAD maintenance confidence | 81 | unknown, confidence level 1-10 |
| Satisfied with VAD outcome (past 3 months) | 82 | unknown, satisfied level 1-10 |
| Method EuroQoL test administered | 77 | Unknown, N/A: not completed, self administered, family member, coordinator |
| Reason KCCQ not completed | 60 | Many: too sick/tired, no time, can’t concentrate, too stressed, N/A (completed) |
| HF limitation: dressing | 81 | Unknown, not limited-extremely limited (6 levels) |
| HF limitation: bathing | 81 | Unknown, not limited-extremely limited (6 levels) |
| HF limitation: housework | 81 | Unknown, not limited-extremely limited (6 levels) |
| HF limitation: stairs | 81 | Unknown, not limited-extremely limited (6 levels) |
| HF limitation: jogging | 81 | Unknown, not limited-extremely limited (6 levels) |
| KCCQ: HF symptoms changed | 81 | Unknown, much worse-much better (6 levels) |
| KCCQ: swelling | 81 | Unknown, much worse-much better (6 levels) |
| KCCQ: swelling bothering | 81 | Unknown, not bothersome-extremely bothersome (6 levels) |
| KCCQ: fatigue | 81 | Unknown, not fatigued-several times a day (6 levels) |
| KCCQ: fatigue bothering | 81 | Unknown, not bothersome-extremely bothersome (6 levels) |
| KCCQ: shortness of breath | 81 | Unknown, no shortness of breath-several times a day (6 levels) |
| KCCQ: shortness of breath bothering | 81 | Unknown, not bothersome-extremely bothersome (6 levels) |
| KCCQ: shortness of breath while sleeping | 81 | Unknown, no shortness of breath-several times a night (6 levels) |
| KCCQ: HF symptoms worsen | 81 | Unknown, much worse-much better (6 levels) |
| KCCQ: patient understands symptoms | 81 | Unknown, never-always (5 levels) |
| KCCQ: enjoyment of life | 81 | Unknown, never-always (5 levels) |
| KCCQ: accepting life with HF | 81 | Unknown, never-always (5 levels) |
| KCCQ: discouraged due to HF | 81 | Unknown, never-always (5 levels) |
| KCCQ: HF affects lifestyle hobbies | 81 | Unknown, does not limit-severely limits (6 levels) |
| KCCQ: HF affects lifestyle chores | 81 | Unknown, does not limit-severely limits (6 levels) |
| KCCQ: HF affects lifestyle visiting family/friends | 81 | Unknown, does not limit-severely limits (6 levels) |
| KCCQ: HF affects lifestyle intimate | 81 | Unknown, does not limit-severely limits (6 levels) |
| KCCQ: HF affects lifestyle walk 1 block | 81 | Unknown, does not limit-severely limits (6 levels) |
| Method KCCQ administered | 81 | Unknown, N/A: not completed, self administered, family member, coordinator |
| Trail making time | 56 | Not completed, <100, 100-160, >160 |
